# Assessment of life support skills of resident dentists using OSCE: cross-sectional survey

**DOI:** 10.1186/s12909-022-03775-z

**Published:** 2022-10-07

**Authors:** Fan Yang, Chen Zheng, Tianer Zhu, Denghui Zhang

**Affiliations:** Stomatology Hospital, School of Stomatology, Zhejiang University School of Medicine, Zhejiang Provincial Clinical Research Center for Oral Diseases, Key Laboratory of Oral Biomedical Research of Zhejiang Province, Cancer Center of Zhejiang University, Hangzhou, 310006 China

**Keywords:** Medical Emergencies, Assessment, Objective Structured Clinical Examination (OSCE)

## Abstract

**Background:**

The aim of this cross-sectional survey was to apply the Objective Structured Clinical Examination (OSCE) to evaluate the cardiopulmonary resuscitation (CPR) and endotracheal intubation skills of resident dentists for stage assessment in standardized training.

**Methods:**

A total of 146 third-year resident dentists were recruited and randomly assigned to perform either CPR or endotracheal intubation. Their performance was scored by experienced anesthesiologists with standardized scoring criteria. Participants were also asked to rated their self-assessed competence, willingness, and perceptions on training status using Likert-type scales in a questionnaire. Student’s ttest was applied to compare scores for CPR and endotracheal intubation performed by resident dentists with different characteristics. The results of the questionnaires were analyzed and visualized by the R package ‘Likert’. Significance was set at the *P* < 0.05 level.

**Results:**

The mean OSCE score for endotracheal intubation (59.1 ± 12.5) was lower than that of CPR (72.4 ± 8.8). Participants with Master’s degrees scored higher than those with Bachelor’s degrees and Doctor of Philosophy (PhD) degrees in the assessment of CPR and endotracheal intubation. Low scores of self-assessed competence and willingness were observed, especially for endotracheal intubation. Resident dentists showed poor satisfaction on training volume and frequency of CPR and endotracheal intubation.

**Conclusion:**

Resident dentists showed poor performance on CPR and endotracheal intubation assessed by the OSCE. Relatively low self-assessed competence and willingness were reported in endotracheal intubation. The medical emergency curriculum for resident dentists should be more consistent and standardized to help resident dentists enhance the proficiency of life support skills.

**Supplementary Information:**

The online version contains supplementary material available at 10.1186/s12909-022-03775-z.

## Introduction

Every dentist may be encountered with a medical emergency in lifetime [1]. For example, anaphylactic shock caused by a local anesthetic allergy, sudden cardiorespiratory arrest during surgery, local anesthetic poisoning, syncope, asphyxia and other emergencies may occur in daily dental practice [2]. In these emergencies, delivering timely and appropriate emergency treatment such as cardiopulmonary resuscitation (CPR) and endotracheal intubation could save lives.

CPR and endotracheal intubation are essential parts of life support measures [3]*.* CPR can restore spontaneous breathing and circulation [4], and endotracheal intubation can protect and maintain the airway. Although the importance of CPR and endotracheal intubation is disputable, studies have reported the relatively poor competency of dental students in CPR and endotracheal intubation [5].

Standardized training for resident dentists is an important process for postgraduate education. Therefore, incorporation of medical emergency training course to standard dental residents training curricula could increase perceived competence in implementing lifu support skills and improve performance of these skills [6]. Moreover, evaluation method to assess residents’ ability to diagnose and manage medical emergencies is needed in dental education [7].

As medical emergencies do occur in the dental treatment process, there is a need for improved teaching of life support skills for dental education in standardized training. Dentists are also expected to be able to apply cardiopulmonary resuscitation techniques during medical emergencies [8]. However, studies have reported increased mortality and morbidity in both prehospital settings and interhospital transfers caused by poor airway care [9, 10]. Junior medical staff reported deficient undergraduate training in emergency procedures, especially airway management. They also showed more interest in gaining more experience in resuscitation and endotracheal intubation as undergraduates [11].

Having been introduced to assess clinical skills of medical students in 1979 [7], the Objective Structured Clinical Examination (OSCE)is a summative examination form to evaluate the clinical competence of students majoring in health professions. It is performance-based testing, which consists of multistation clinical skill examinations [12].

One of the most distinguishing advantages of the OSCE is the directness and objectiveness of clinical skill assessments. Therefore, the OSCE is widely applied in residency training evaluating system in Western countries [13]. The OSCE was also applied to assess the clinical skills of resident dentists in standardized training for resident dentists [12] and assess life support skills among medical students [14]. The assessment criteria were a written checklist of several predetermined items. These criteria were set by the clinical disciplines and were based on clinical evaluation rubrics of the Institute of Dentistry. Each station uses a 100%-based system, and the final score was the sum of scores from all the stations.

The aim of this study was to include CPR and endotracheal intubation as part of the OSCE for stage assessments in standardized training to evaluate the life support skills of resident dentists.

## Methods

### IRB Approval

Informed consent was obtained from all subjects. The project was approved by the Ethics Committee of Stomatology Hospital, School of Stomatology, Zhejiang University School of Medicine (No. 2021–77(R)).

### Participants

This cross-sectional study included all the 146third-year resident dentists (65 males and 81 females) who received a stage assessment in standardized training in 2021, organized by the Department of Education of the Stomatology Hospital, Medical School of Zhejiang University and conducted at the Clinical Simulation Center of Zhejiang University.

All resident dentists had a dentist license and received three training sessions on CPR and endotracheal intubation uniformly organized by the Department of Education of the Stomatology Hospital one month before the assessment, before which no resident dentists had received any training on CPR or endotracheal intubation in standardized training.

### The practical exam

The practical exam included CPR and endotracheal intubation both using mannequin simulators. All the resident dentists were randomly assigned to perform one of the above two life support skills. There was no crossover of participants in either group. The random assignment scheme was created from a table of random numbers. Anesthetists are undoubtedly airway experts, so anesthetists with at least 10 years of experience and titled with deputy chief physician were asked to be the examiners in the assessment. All participants independently completed the practice exam.

In the practical exam on CPR, a participant encounters a patient who is experiencing sudden cardiac arrest outdoors. The participant should ensure safety, check the patient’s situation, perform CPR on the mannequin simulator, judge the resuscitation effect, and answer a theoretical question about CPR raised by the examiners. In the practical exam on endotracheal intubation, a participant encounters a patient needing endotracheal intubation due to a dental emergency in the hospital. The participant should prepare the equipment and patient, preoxygenate before intubation, perform endotracheal intubation on the mannequin simulator, confirm tube placement, and answer a theoretical question about endotracheal intubation raised by the examiners.

### Assessments

Skill checklists of the OSCE were developed for resident dentists across the criteria required for the performance of CPR and endotracheal intubation (Table S1, S2).Using a consensus-building process with three anesthesia staff members experienced in the assessment and education research, we modified the checklists to permit the assessment of CPR and intubation skills. Just two rounds of e-mail discussions were required to reach consensus on the new form. We regard this process as the form of evidence of content validity [15]. We also evaluated interrater reliability using an intraclass correlation coefficient to support the validation of the checklists.

Scores given by two examiners were averaged to be the final score. In the CPR test session, the rate, depth and recoil of the compression were shown on the display connected with the mannequin and the examiners referred to the result as the final score.

The resident dentists were provided with a form for the collection of demographic data and an after-test questionnaire on self-assessed competence, willingness (Table S3, S4) and perception of training status (Table S5) using Likert type scales (scores of 1–5) [16]. Higher scores indicated that participants agreed more with the item. A test–retest method was used to validate the reliability of the questionnaire. All the participants were asked to complete the survey again after 1 week resulting in a Pearson correlation coefficient of 0.86 for questionnaire about CPR and 0.89 for questionnaire about endotracheal intubation.

### Data analysis and definitions

The following results were measured: (1) demographic information of the participating resident dentists; (2) scores (total and for each portion) for CRP and endotracheal intubation exams and (3) self-assessed competence, willingness and perception of training status in response to the questionnaires. Data were managed and analyzed with SPSS 22.0 (SPSS, Chicago, IL). Student’s *t* test was applied to compare the scores (total and for each portion) for the CPR and endotracheal intubation exams completed by resident dentists with different characteristics. Cronbach's α was used to determine the internal consistency of the questionnaires using Likert-type scales. The results of the questionnaires were analyzed and visualized by the *R* package ‘Likert’. Significance was set at the *P* < 0.05 level.

## Results

### Demographic information of the resident dentists receiving the OSCE assessments

A total of 146 resident dentists received the OSCE assessments. All of the participants were assigned to randomly perform either CPR (*n* = 74) or endotracheal intubation (*n* = 72). The demographic information of the assessed participants, including sex, age and the highest educational attainment, is shown in Table 1. There were no statistical difference between two groups on demographic information.

**Table 1:** Demographic information of resident dentists participating in assessment of life support skills using OSCE

	CPR	Endotracheal intubation	Total	Statistics
**Number**	74	72	146	
**Sex**				χ^2^ = 0.0003, *P* = 0.9854
Male	33(44.6%)	32(44.4%)	65(44.5%)	
Female	41(55.4%)	40(55.6%)	81(55.5%)	
**Age**	26.60 ± 1.44	26.77 ± 1.38	26.68 ± 1.41	t = 0.7280, *P* = 0.4678
**Education**				χ^2^ = 0.5274, *P* = 0.7682
Bachelor	36(48.6%)	38(52.8%)	74(50.7%)	
Master	32(43.2%)	27(37.5%)	59(40.4%)	
PhD	6(8.1%)	7(9.7%)	13(8.9%)	

### OSCE-assessed scores for CPR and endotracheal intubation

**Fig. 1:**
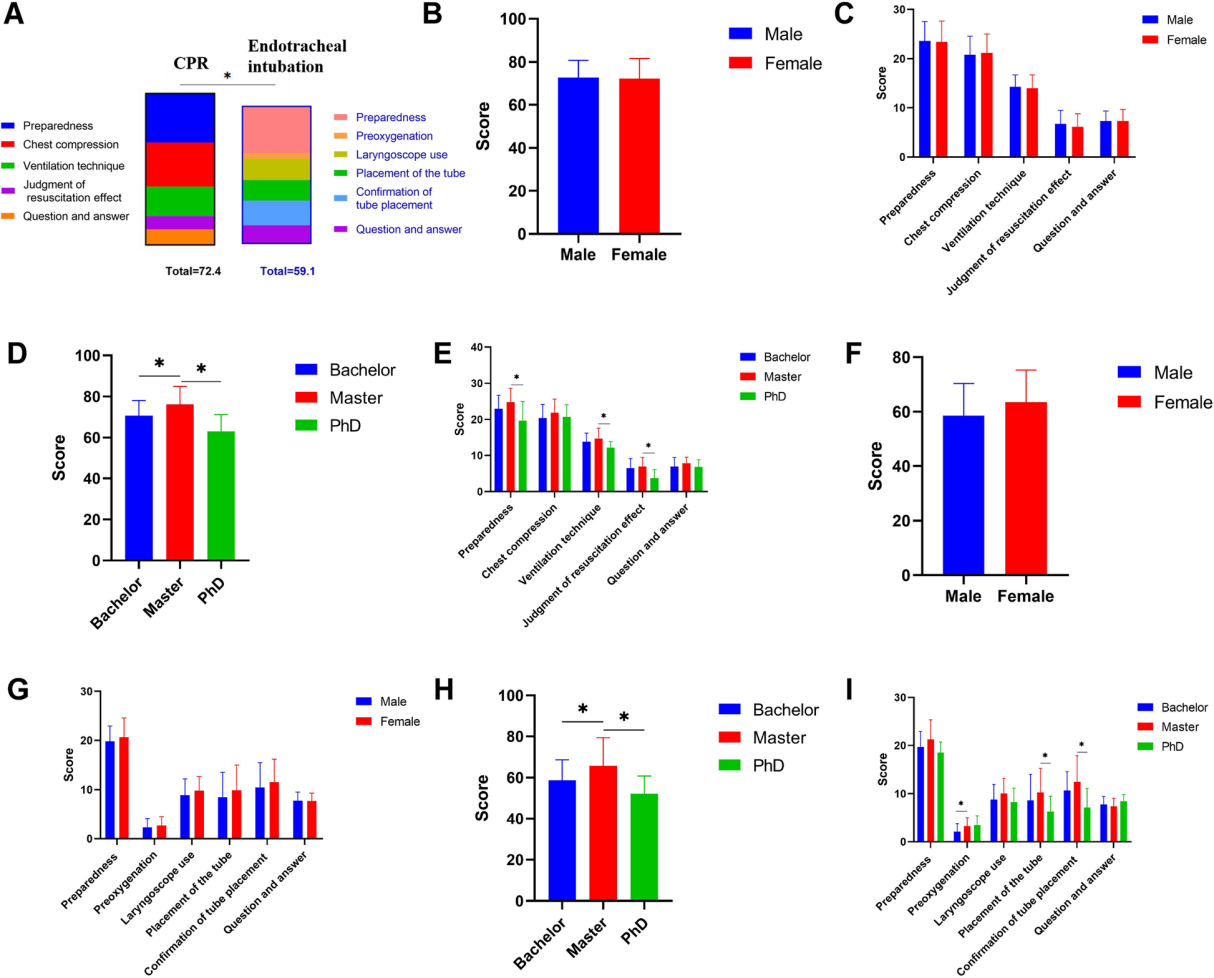
OSCE-assessed scores of CPR and endotracheal intubation. **A** The total score of CPR and endotracheal intubation. **B** The total score of CPR in male and female resident dentists. **C** The CPR scores of each block in male and female resident dentists. **D** The total score of CPR in resident dentists with different education attainment. **E** The CPR scores of each block in resident dentists with different education attainment. **F** The total score of endotracheal intubation in male and female resident dentists. **G** The endotracheal intubation scores of each block in male and female resident dentists. **H** The total score of endotracheal intubation in resident dentists with different education attainment. **I** The endotracheal intubation scores of each block in resident dentists with different education attainment. *: *P* < 0.05

Seventy-four resident dentists performed CPR and 76 resident dentists performed endotracheal intubation. The interrater reliability across all ratings of endotracheal intubation skills (ICC = 0.86) and CPR (ICC = 0.83) was strong. The mean OSCE score for CPR was 72.4 ± 8.8 (highest possible score of 100), with other scores as follows:23.5 ± 4.1 for preparedness for delivering CPR (highest possible score of 30);21.1 ± 3.8 for chest compression (highest possible score of 30);14.1 ± 2.6 for ventilation technique (highest possible score of 20);6.4 ± 2.7 in judgment of resuscitation effect t(highest possible score of 10); and7.3 ± 2.2 in question and answer portion (highest possible score of 10) (Fig. 1A). For resident dentists of different sexes, there was no significant difference between the scores of males and females in total (72.7 ± 8.1 vs. 72.1 ± 9.5, *P* > 0.05) (Fig. 1B) and on the respective part (*P* > 0.05) (Fig. 1C). However, educational attainment has significant effects on OSCE-assessed CPR scores. Participants with master’s degrees scored higher than those with bachelor’s degrees (76.1 ± 8.8 vs. 70.7 ± 7.4, *P* < 0.05) and even those with PhD degrees (76.1 ± 8.8 vs. 63.0 ± 8.2, *P* < 0.05) (Fig. 1D). No significant difference was observed between participants with a master’s degree and those with a bachelor’s degree (*P* > 0.05), while significant differences were observed between participants with a master’s degree and those with a PhD degree for “preparedness in delivering CPR”, “ventilation technique” and “judgment of resuscitation effect” (*P* < 0.05) (Fig. 1E).

The mean OSCE score for endotracheal intubation was lower than that of CPR (*P* < 0.05), which was 59.1 ± 12.5 (highest possible score 100), with the other scores as follows: 20.1 ± 3.5 for preparedness of equipment and patients (highest possible score of 30); 2.5 ± 1.7 for preoxygenation before every intubation (highest possible score of 5); 9.2 ± 3.1 for using the laryngoscope correctly (highest possible score of 15); 8.9 ± 5.1 for successful placement of the tube into the trachea (highest possible score of 20); 10.7 ± 5.0 for confirmation of tube placement (highest possible score of 20); and 7.7 ± 1.7 for the question and answer portion (highest possible score of 10) (Fig. 1A). For resident dentists of different sexes, despite no significant difference between the scores of males and females in total (58.6 ± 11.7 vs. 63.5 ± 11.8, *P* > 0.05) (Fig. 1F) and on the respective parts (*P* > 0.05) (Fig. 1G), female resident dentists scored higher than male resident dentists. Meanwhile, educational attainment had significant effects on OSCE-assessed scores for endotracheal intubation. Participants with master’s degrees scored more than those with bachelor’s degrees (65.6 ± 13.8 vs. 58.7 ± 9.9, *P* < 0.05) and PhD degrees (65.6 ± 13.8 vs. 52.1 ± 8.6, *P* < 0.05) overall (Fig. 1H). A significant difference was only observed between participants with master’s degrees and those with bachelor’s degrees only on the “preoxygenation” part (*P* < 0.05), while significant differences were observed between participants with master’s degrees and those with PhD degrees in the “placement of the tube” and “confirmation of tube placement” (*P* < 0.05) (Fig. 1I).

### Self-assessed competence of CPR and endotracheal intubation

Of the 146 resident dentists receiving the OSCE assessments, 119 (82%) responded to the questionnaire, while 4 of them were excluded because of missing values in relation to the analysis. A total of 115 questionnaires were included in the survey. Of the 115 resident dentists who responded to the questionnaires, 56 performed CPR, and 59 performed endotracheal intubation for assessment. The internal consistency of the questionnaire was evaluated by Cronbach's α coefficient and was 0.74 and 0.78, respectively, for resident dentists delivering CPR or endotracheal intubation.

**Fig. 2:**
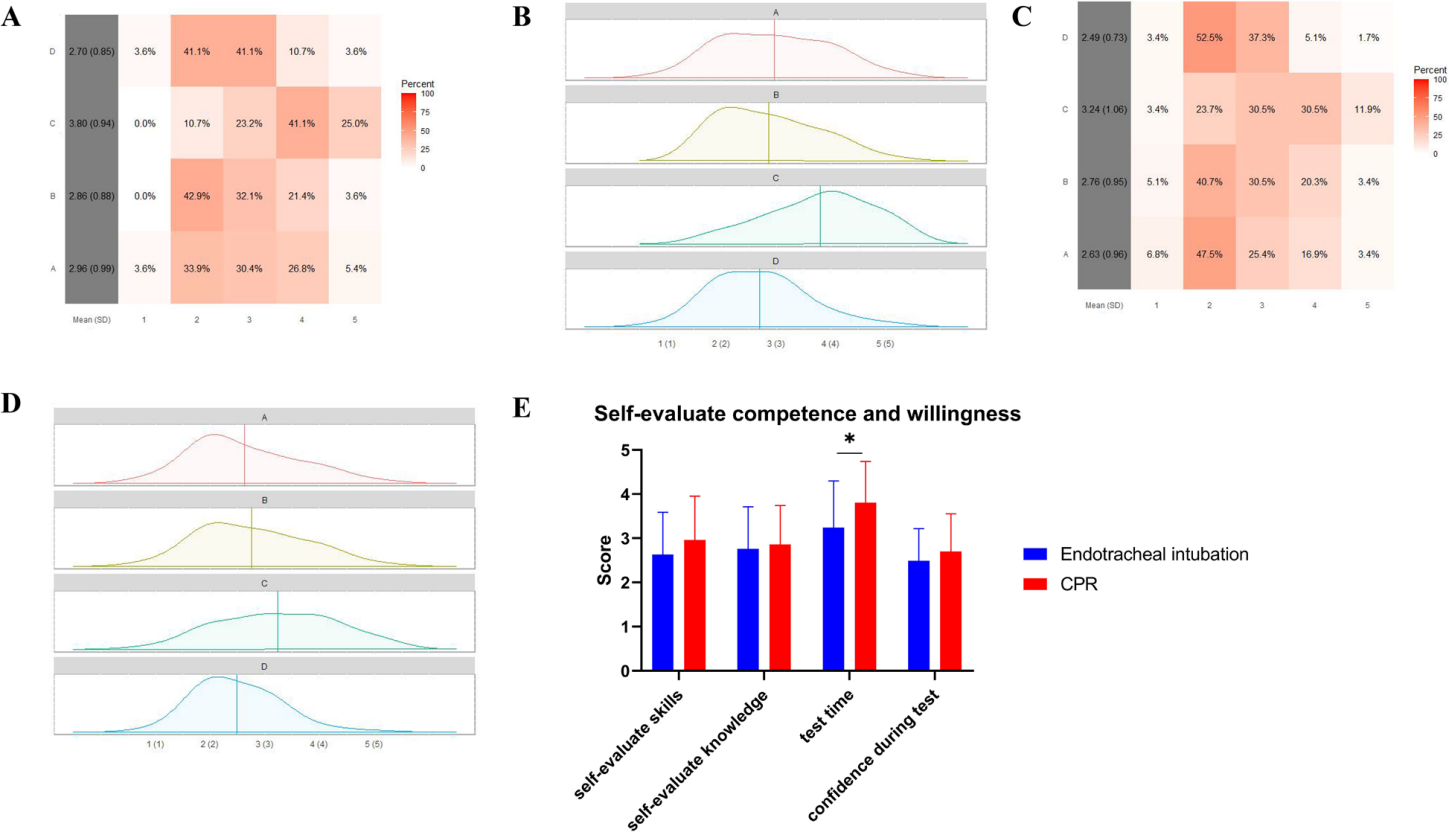
Visualization and comparison of questionnaire results on self-assess competence of CPR and endotracheal intubation. 4 questions were as followed for resident dentists delivering CPR: A, I have a good command of skills of CPR. B, I have a good command of knowledge of CPR. C, I have enough time to finish CPR in the test. D, I have confidence performing CPR during test. 4 questions were as followed for resident dentists delivering endotracheal intubation: A, I have a good command of skills of endotracheal intubation. B, I have a good command of knowledge of endotracheal intubation. C, I have enough time to finish endotracheal intubation in the test. D, I have confidence performing endotracheal intubation during test. 5 options were as followed: 1, strongly disagree. 2, disagree. 3, not sure. 4, agree. 5, strongly agree. **A** Heatmap of questionnaire results on self-assess competence of CPR. **B** Density map of questionnaire results on self-assess competence of CPR. **C** Heatmap of questionnaire results on self-assess competence of endotracheal intubation. **D** Density map of questionnaire results on self-assess competence of endotracheal intubation. **E** Comparison of questionnaire results between CPR and endotracheal intubation. *: *P* < 0.05

In terms of self-assessed competence of CPR, the mean scores of the 4 corresponding questions were 2.96 ± 0.99 (I had a good command of the skills for CPR; highest possible score of 5), 2.86 ± 0.88 (I had a good command of the knowledge for CPR; highest possible score of 5), 3.80 ± 0.94 (I had enough time to finish CPR in the test; highest possible score of 5), and 2.70 ± 0.85 (I had confidence in performing CPR during the test; highest possible score of 5), respectively (Fig. 2A, B).

In terms of self-assessed competence of endotracheal intubation, the mean scores of the 5 corresponding questions were 2.63 ± 0.96 (I had a good command of the skills for endotracheal intubation; highest possible score of 5), 2.76 ± 0.95 (I had a good command of the knowledge for endotracheal intubation; highest possible score of 5), 3.24 ± 1.06 (I had enough time to finish endotracheal intubation in the test; highest possible score of 5), and 2.49 ± 0.73 (I had confidence in performing endotracheal intubation during the test; highest possible score of 5) (Fig. 2C, D).

Resident dentists who performed CPR responded with more scores than those who performed endotracheal intubation on the test (*P* = 0.003). Self-assessed skills (*P* = 0.07) and confidence during the test (*P* = 0.16) tended to be higher in the CPR group than in the endotracheal intubation group, although the difference did not reach statistical significance (Fig. 2E). However, self-assessed knowledge did not differ between the CPR and endotracheal intubation groups (*P* = 0.56) (Fig. 2E).

### Willingness to clinically perform CPR and endotracheal intubation

**Fig. 3:**
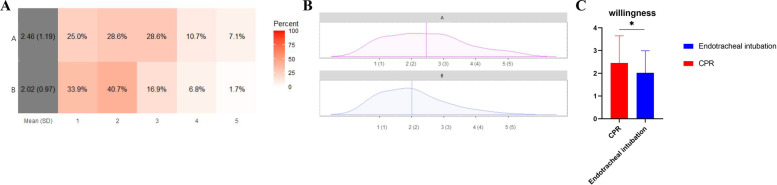
Visualization and comparison of questionnaire results on willingness to perform CPR and endotracheal intubation clinically. 5 options were as followed: 1, strongly disagree. 2, disagree. 3, not sure. 4, agree. 5, strongly agree. **A** Heatmap of questionnaire results on willingness of CPR. **B** Density map of questionnaire results on willingness of CPR. **C** Comparison of questionnaire results between CPR and endotracheal intubation. *: *P* < 0.05

In terms of willingness to perform CPR and endotracheal intubation, the mean scores were 2.46 ± 1.19 (I am willing to perform CPR clinically; highest possible score of 5) and 2.02 ± 0.97 (I am willing to perform endotracheal intubation clinically; highest possible score of 5) (Fig. 3A, B).

Resident dentists performing CPR responded with more willingness scores than those performing endotracheal intubation (*P* = 0.03) (Fig. 3C).

### Perceptions on training status of CPR and endotracheal intubation

**Fig. 4:**
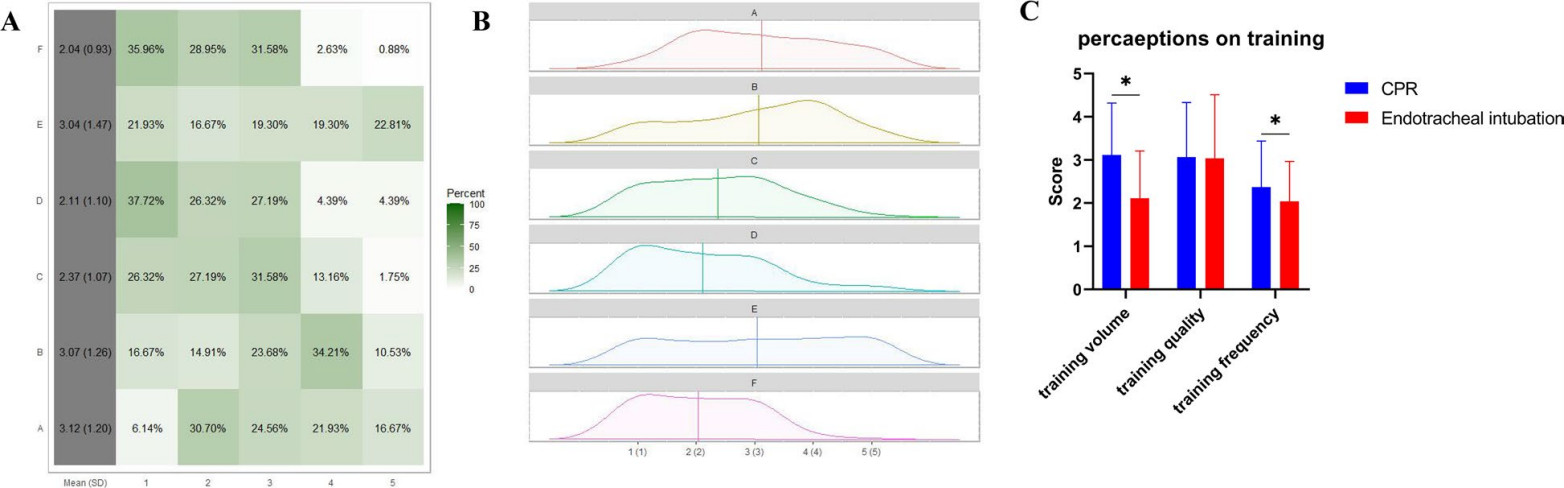
Visualization and comparison of questionnaire results on perceptions on training status of CPR and endotracheal intubation. 6 questions were as followed: A, I have received enough training of CPR during standardized training. B, the training of CPR during standardized training is of high quality. C, frequency of CPR training is suitable. D, I have received enough training of endotracheal intubation during standardized training. E, the training of endotracheal intubation during standardized training is of high quality. F, Frequency of each endotracheal intubation training is suitable. **A** Heatmap of questionnaire results on perceptions on training status of CPR and endotracheal intubation. **B** Density map of questionnaire results on perceptions on training status of CPR and endotracheal intubation. **C** Comparison of questionnaire results on perceptions on training status between CPR and endotracheal intubation. *: *P* < 0.05

A total of 115 resident dentists answered 6 questions related to perception of their training status of CPR and endotracheal intubation (Fig. 4A, B).

For CPR, the mean scores of the 3 corresponding questions were 3.12 ± 1.20 (I have received enough CPR training during standardized training; highest possible score of 5), 3.07 ± 1.26 (The training for CPR during standardized training is of high quality; highest possible score of 5), and 2.37 ± 1.07 (The frequency of CPR training is suitable; highest possible score of 5).

For endotracheal intubation, the mean scores of the 3 corresponding questions were 2.11 ± 1.10 (I have received enough training for endotracheal intubation during the standardized training; highest possible score of 5), 3.04 ± 1.47 (The training for endotracheal intubation during standardized training is of high quality; highest possible score of 5), and 3.12 ± 1.20 (The frequency of each endotracheal intubation training was suitable; highest possible score of 5), which were all relatively lower than the perceptions of CPR training, except for the quality (Fig. 4C).

## Discussion

To our knowledge, this is the first paper to investigate resident dentists’ OSCE-assessed competency and perceptions of life support skills. These findings indicated that resident dentists lacked emergency experiences and had limited competency and confidence in performing life support skills.

According to the assessment results, resident dentists had poor proficiency in life support skills, especially endotracheal intubation. Successful endotracheal intubation has no standard definition. Most inexperienced students or residents would regard the placement of the tracheal tube as the most important step in defining a successful intubation. However, a prolonged intubation attempt, delaying the initiation of oxygenation and ventilation could be caused by ignoring checking equipment before intubation. Unrecognized esophageal intubation is considered the most serious complication of attempted endotracheal intubations, so failure to confirm tracheal tube placement is very dangerous [17]. Serious complications could be caused by failure to deliver the accompanying steps. Therefore, correct endotracheal intubation was defined by the performance in five main sessions in our study: preparedness of equipment and patients, preoxygenation before every intubation, using the laryngoscope correctly, successful placement of the tube into the trachea and confirmation of tube placement. Poor performances were observed in preoxygenation, laryngoscope use, placement of the trachea tube and confirmation of tube placement in the OSCE in the present study. Resident dentists often forgot the preoxygenation step, especially after a failure attempt of intubation, and laryngoscopes were usually placed too deep to expose the glottis. In the process of intubation, failed intubation often occurred and poor proficiency was observed in most of the resident dentists.

The effects of sex and educational attainment on endotracheal intubation performance were analyzed in our study. The results showed that sex had no significant effect on the OSCE score for endotracheal intubation either in total or in each block. It was notable that resident dentists with PhD degrees had the lowest scores in endotracheal intubation performance. We speculated that resident dentists with PhD degrees majored in subspecialties such as endodontics and periodontology and undertook too many scientific tasks during standardized training, so they might have spent less time learning about emergency medicine than those with master or bachelor degrees. Academic outcomes, but not clinical competence, are often the most important index of judging the competence of Chinese dentists with higher academic degrees and determining their careers and promotions. Hence, graduates with PhD degrees face higher demands for academic research than for clinical training. Therefore, more academic achievement must be attained even at the cost of essential clinical training time [18], leading to the imbalance of academic research and clinical training.

Educational attainment had a significant effect on the OSCE scores for endotracheal intubation in total score and the blocks of preoxygenation, placement of the tube and confirmation of tube placement. The grades from highest to lowest were for those with master degrees, bachelor degrees, and PhD degrees, respectively. In another study conducted in China, graduates with PhD degrees felt the dilemma in balancing academic research and clinical training [19]. In addition, the limited sample size might have influenced the reliability of the conclusions. Above all, more attention should be paid to resident dentists with PhD degrees concerning the life support training.

Although resident dentists scored higher in CPR than endotracheal intubation, there were still several particular concerns in the CPR assessment. In terms of the assessment of patient conditions, some residents ignored the observation of thoracic fluctuation and wasted too much time before beginning compression. In the compression session, many residents could not maintain high quality compression throughout the 5 cycles mainly because of lack of endurance, leading to a faster rate, shallower depth and inadequate recoil. In terms of ventilation, some residents forgot to pinch the patient’s nostril shut, which might lead to ventilation failure. Regarding the judgment of the resuscitation effect, many residents forgot to observe spontaneous breathing and the time spent on checking the carotid pulse was usually too short. Lacking enough and regular training might account for the poor acquisition of life support skills. In line with the endotracheal intubation, resident dentists with PhD degrees had the lowest CPR performance scores, while those with Master degrees had the highest CPR performance scores. Therefore, special attention needs to be paid to the life support skills training of resident dentists with PhD degrees.

According to the survey, resident dentists responded low confidence in delivering life support skills in medical emergencies, especially endotracheal intubation. The willingness to perform endotracheal intubation was lower than that of CPR, which was in line with the results of the self-assessed competence and skill assessment scores. A previous study showed that well-prepared students had a high self-evaluation of their life support skills, which was even higher than their actual performance [20], while another study found that confidence in performing life support skills was lower than that in performance for students receiving training 2 years before testing [21]. Therefore, regular training helped to improve the confidence of delivering CPR and endotracheal intubation in emergency situations. However, dental residents were reported to have low scores in perceptions on training of life support skills in terms of volume and frequency, especially endotracheal intubation in the present study. In turn, a lack of training and poor proficiency of life support skills could account for low confidence and even decrease the willingness to perform life support skills in emergencies.

CPR and endotracheal intubation are considered fundamental life support skills in medical emergencies [3].Therefore, we should search for effective methods to improve the acquisition of the life support skills by resident dentists. Furthermore, previous studies suggested that competence and self-assessed confidence in performing CPR and endotracheal intubation were decreased significantly and quite rapidly [22, 23]; therefore, the medical emergency curriculum for resident dentists should be more consistent and standardized to help residents enhance their proficiency and retention of life support skills.

There were limitations of this study. We did not include laryngeal mask airway (LMA) in the study since it is important part of ACLS, which should be included in further study. And we did not record time consumed in endotracheal intubation. Prolonged intubation may increase the risk of desaturation, and aspiration and affect the performance of CPR. Despite the importance of intubation time, we felt that if timing was included in assessment, the residents would overemphasize the need to ‘shove the tube in’ urgently so they might be too stressed to perform complete endotracheal intubation. In addition, we did not record the time because we cared more about assessing the residents’ competency delivering a complete and correct endotracheal intubation. Furthermore, all residents were required to preoxygenate the patients for 2 min using a bag and mask ventilation before every intubation attempt, which would give them enough time to perform the intubation. Secondly, the number of participants with PhD degrees is small due to limited number of resident dentists who have got a PhD degree and the real life experiences might contribute to the better performance by maters level resident. Another limitation was the use of mannequin simulators instead of patients for both CPR and endotracheal intubation. It has been reported that experience gained on mannequin simulators may not be translated into successful life support skills for patients [24]. However, previous findings suggested that the skills gained on mannequin simulators could be translated into successful CPR and endotracheal intubations for patients [25, 26]. Furthermore, mistakes were allowed without risk to patients’ safety and the procedure could be performed slowly or stopped for teaching and repeated attempts. Different emergency situations could be created and different equipment and techniques could also be applied in one situation. Resident dentists could practice as often as possible so many attempts could be made in quick succession. Additionally, practices and tests could be scheduled according to the training schedules of the residents. In terms of the precision of research, the environment could be controlled to limit the distractions and cognitive load with the application of mannequin simulators, making the results more reliable. Last but not least, resident dentists appreciated being skilled and confident in life support skills performed on mannequin before attempting them on a real patient. Consequently, we believe that the findings are reasonable for assessing the life support skills of resident dentists.

## Conclusion

Resident dentists showed poor performance in CPR and endotracheal intubation assessed by the OSCE. Relatively low self-assessed competence and willingness were reported, especially in endotracheal intubation. The medical emergency curriculum for resident dentists should be more consistent and standardized to help resident dentists enhance their proficiency of life support skills.

## Supplementary Information

**Additional file 1: Table S1.** OSCE criteria scores of CPR for stage assessment in standardized training.

**Additional file 2: Table S2.** OSCE criteria scores of endotracheal intubation for stage assessment in standardized training.

**Additional file 3: Table S3.** Questionnaire on self-assess competence and willingness of CPR.

**Additional file 4: Table S4.** Questionnaire on self-assess competence and willingness of endotracheal intubation.

**Additional file 5: Table S5.** Questionnaire on perception on training of first-aid skills during standardized training.

## Data Availability

All data are available in the main text or the supplementary materials.

## Abbreviations

ACLS: Advanced cardiac life support
CPR: Cardiopulmonary resuscitation
OSCE: Objective Structured Clinical Examination
PhD: Doctor of Philosophy
