# Supplementary material for: Assessment of life support skills of resident dentists using OSCE: cross-sectional survey
Source: BMC Med Educ. 2022 Oct 7;22:710. doi: 10.1186/s12909-022-03775-z (PMC9541086; doi:10.1186/s12909-022-03775-z)
Supplement: Supplementary file 1 — Additional file 1: Table S1. OSCE criteria scores of CPR for stage assessment in standardized training. [file 12909_2022_3775_MOESM1_ESM.docx]

Table S1. OSCE criteria scores of CPR for stage assessment in standardized training

| Items (score) | Criterion | Total score |
| --- | --- | --- |
| Preparedness for delivering CPR (30) | Ensure safety | 10 |
|  | check patient consciousness, call for help, activate the emergency management system (EMS), obtain automated external defibrillator (AED) | 10 |
|  | Check the patient's breathing and sense the carotid pulse for 5-10 seconds | 10 |
| Chest compression (30) | Place patient in supine position on hard ground | 5 |
|  | Press the lower half of the sternum, slightly lower than the midpoint of the bilateral nipple line | 5 |
|  | Cross hands, keep upper arms vertical, don't bend elbows | 5 |
|  | Perform chest compressions at a rate of 100-120 per minute | 5 |
|  | Press at depth of 5- 6cm | 5 |
|  | Make the chest recoil adequately after each compression | 5 |
| Ventilation technique (20) | Cease to deliver 2 breaths after each 30 compressions | 5 |
|  | Deliver 2 breaths for no more than 10 seconds | 5 |
|  | Pinch nostril shut, cover the mannequin’s mouth and seal it | 5 |
|  | Perform effective artificial respiration to make chest rise， avoid hyperventilation | 5 |
| Judgment of resuscitation effect (10) | Check carotid pulse and spontaneous breathing after 5 cycles | 10 |
| Question and answer (10) | Answer a theoretical question about CPR | 10 |
