# Supplementary material for: Assessment of life support skills of resident dentists using OSCE: cross-sectional survey
Source: BMC Med Educ. 2022 Oct 7;22:710. doi: 10.1186/s12909-022-03775-z (PMC9541086; doi:10.1186/s12909-022-03775-z)
Supplement: Supplementary file 2 — Additional file 2: Table S2. OSCE criteria scores of endotracheal intubation for stage assessment in standardized training. [file 12909_2022_3775_MOESM2_ESM.docx]

Table S2. OSCE criteria scores of endotracheal intubation for stage assessment in standardized training

| Items (score) | Criterion | Total score |
| --- | --- | --- |
| Preparedness of equipment and patients (30) | Wash hands, wear hat, mask, gloves | 5 |
|  | Verify patient information, get informed consent, determine the possibility of difficult intubation | 5 |
|  | Select suitable endotracheal tube， check the air bag， prepare a laryngoscope and check the light | 5 |
|  | Prepare other equipment: a sputum aspirator, a bag-and-mask, a guide wire, a stethoscope | 5 |
|  | Position patient on supine position，make the mouth, pharynx, and trachea aligned | 10 |
| Preoxygenation before every intubation (5) | Preoxygenate patients for 2 minutes using bag-and-mask | 5 |
| Using the laryngoscope correctly (15) | Held the mouth open， remove the secretion and foreign matter | 5 |
|  | Insert the laryngoscope with left hand, press the base of the tongue, expose the glottis | 10 |
| Successful placement of the tube into the trachea (20) | slowly reach suitable depth | 10 |
|  | pull out the guide wire, place dental pad, remove the laryngoscope | 10 |
| confirmation of tube placement (20) | Deliver gas into the air bag, deliver oxygen using a BVM | 5 |
|  | observe the fluctuation of the chest, listen to both lungs for breath sound using a stethoscope | 10 |
|  | Fix the endotracheal tube and dental pad with tape | 5 |
| Question and answer (10) | Answer a theoretical question about endotracheal intubation | 10 |
