# Supplementary material for: Assessment of life support skills of resident dentists using OSCE: cross-sectional survey
Source: BMC Med Educ. 2022 Oct 7;22:710. doi: 10.1186/s12909-022-03775-z (PMC9541086; doi:10.1186/s12909-022-03775-z)
Supplement: Supplementary file 4 — Additional file 4: Table S4. Questionnaire on self-assess competence and willingness of endotracheal intubation. [file 12909_2022_3775_MOESM4_ESM.docx]

Table S4. Questionnaire on self-assess competence and willingness of endotracheal intubation.

| Option (score) | strongly disagree（1） | Disagree（2） | Not sure（3） | Agree（4） | strongly agree（5） |
| --- | --- | --- | --- | --- | --- |
| I have a good command of skills of endotracheal intubation |  |  |  |  |  |
| I have a good command of knowledge of endotracheal intubation |  |  |  |  |  |
| I have enough time to finish endotracheal intubation in the test |  |  |  |  |  |
| I have confidence performing endotracheal intubation during test |  |  |  |  |  |
| I am willing to perform endotracheal intubation clinically |  |  |  |  |  |
