# Supplementary material for: Assessment of life support skills of resident dentists using OSCE: cross-sectional survey
Source: BMC Med Educ. 2022 Oct 7;22:710. doi: 10.1186/s12909-022-03775-z (PMC9541086; doi:10.1186/s12909-022-03775-z)
Supplement: Supplementary file 5 — Additional file 5: Table S5. Questionnaire on perception on training of first-aid skills during standardized training. [file 12909_2022_3775_MOESM5_ESM.docx]

Table S5. Questionnaire on perception on training of first-aid skills during standardized training

| Option (score) | strongly disagree（1） | Disagree（2） | Not sure（3） | Agree（4） | strongly agree（5） |
| --- | --- | --- | --- | --- | --- |
| I have received enough training of CPR during standardized training |  |  |  |  |  |
| The training of CPR during standardized training is of high quality. |  |  |  |  |  |
| Frequency of CPR training is suitable. |  |  |  |  |  |
| I have received enough training of endotracheal intubation during standardized training |  |  |  |  |  |
| The training of endotracheal intubation during standardized training is of high quality. |  |  |  |  |  |
| Frequency of each endotracheal intubation training is suitable. |  |  |  |  |  |
